# Sensory Features, Liking and Emotions of Consumers towards Classical, Molecular and Note by Note Foods

**DOI:** 10.3390/foods10010133

**Published:** 2021-01-10

**Authors:** Artur Głuchowski, Ewa Czarniecka-Skubina, Eliza Kostyra, Grażyna Wasiak-Zys, Kacper Bylinka

**Affiliations:** 1 Department of Food Gastronomy and Food Hygiene, Institute of Human Nutrition Sciences, Warsaw University of Life Sciences (WULS), 02-787 Warsaw, Poland; artur_gluchowski@sggw.edu.pl (A.G.); kacperbylinka@gmail.com (K.B.); 2 Department of Functional and Organic Food, Institute of Human Nutrition Sciences, Warsaw University of Life Sciences (WULS), 02-787 Warsaw, Poland; eliza_kostyra@sggw.edu.pl (E.K.); grazyna_wasiak_zys@sggw.edu.pl (G.W.-Z.)

**Keywords:** new product development, molecular and Note by Note products, classical dishes, sensory profiling, emotions, CATA, consumer research

## Abstract

Modern cuisine served at top-end restaurants attempts to attract customers, who increasingly demand new flavor, pleasure and fun. The materials were six dishes prepared using lemon or tomatoes and made in the traditional (classical), molecular and Note by Note (NbN) versions. The study explores sensory characteristics, consumer liking of key attributes, their declared sensations and emotions, as well as consumers’ facial expressions responding to the dishes. These objectives were investigated by descriptive quantitative analysis and consumer tests. Tests included a 9-point hedonic scale for degree of liking a dish, Check-All-That-Apply (CATA) for declared sensations and FaceReader for facial expressions. The influence of factors associated with consumer attitudes toward new food and willingness to try the dishes in the future were also determined. It was stated that the product profiles represent different sensory characteristics due to the technology of food production and the ingredients used. The food neophobia and consumer innovativeness had a significant (*p* ≤ 0.05) effect on liking. The odor-, flavor-, texture- and overall-liking of the NbN dishes were lower than that of traditional versions but did not vary from scores for molecular samples. The expected liking of NbN dishes was higher than experienced-liking. Traditional and modern products differed in CATA terms. Classical dishes were perceived by consumers as more tasty, traditional and typical while modern cuisine dishes were perceived as more surprising, intriguing, innovative and trendy. Mimic expressions assessment by FaceReader showed similar trends in some emotions in both classical dishes and separate temporal patterns in modern products.

## 1. Introduction

Choice of food is driven by numerous biological, economic, physical and socio-psychological determinants. The changes in global dietary patterns are influenced by various trends and factors. For example, when eating out, consumers do not just want to meet their physiological needs but are in search of food that brings emotional benefits and gives the pleasure of new flavors and tastes [1,2,3]. There are many factors that contribute to food satisfaction for consumers’ eating out experiences. The most common factors include satiation, sensory experience, food variety and quality (nutritional value, origin, healthiness, visual attractiveness, freshness), financial aspects (price/value, promotion), company of friends or family [4,5,6,7,8,9,10,11,12,13,14,15,16,17,18,19,20,21], restaurant service, food presentation, taste of the food and the physical details of the restaurant (e.g., interior colors and design, music and mood) [6,8,16,18,19,20,21].

Molecular cuisine and Note by Note cooking (NbN) are novelties that have appeared in recent years. Molecular cooking is typically defined as producing food in professional kitchens using “new” tools (siphons, evaporators, etc.), ingredients (food additives, surprising texturizers) and methods (sous-vide cooking, flash freezing, etc.) [22]. Modern cuisine uses the “food deconstruction” technique, which consists of preparing a classic dish, with its same ingredients and flavors, and presenting it in a very unconventional and nontraditional manner [23]. Note by Note cuisine is the use of pure compounds or their mixtures in the production of dishes. Compounds are used to design the shape, color, taste, odor, temperature, trigeminal stimulation, texture and nutritional aspects of the developed meal [24,25,26,27]. The concept of modern cuisines is to create avant-garde, unique dishes that surprise customers. Modern restaurants’ dishes should provide a multisensory experience involving emotions, memory, culture [28] and sensory perception, especially by aesthetic visual appearance. It also typically provides rich contrasts of flavors (through, for example, surprising ingredient pairings), textures and temperatures [29,30]. This sensory incongruity and the associated surprise evoke various responses from different people that depend on a consumer’s fear and willingness to try new foods [31].

Consumers still do not trust new food technologies, especially when they do not know their effect on human health and the environment [32]. Acceptance of the modification of traditional food depends on their character that results from the changes. Innovations like diversification of shapes and textures and unexpected combinations of ingredients to create new flavors were perceived as the least acceptable and as the most harmful to the traditional character of food [33].

Success of markets for new food technologies depends on consumers’ behavioral responses and emotional states [34,35] and is primarily associated with sensory properties [36]. Disgust, food neophobia and related features have been recognized as the main barrier to accepting novel food [37], and negative and suspicious consumer attitudes towards food technologies may lead to product failure [34]. Additionally, the preferences of modern consumers for food prepared at catering establishments are contradictory. On the one hand, consumers appreciate the naturalness of food [38], which is perceived as a crucial feature [39]. On the other hand, consumers accept novel technologies like modern cuisine, which contains food additives [40].

The evaluation of molecular and Note by Note meals is not easy because the idea of creating them is based on the constant novelty and uniqueness of dishes. Our results previously revealed a positive attitude of consumers toward molecular dishes, their moderately high sensory-experience ratings, and openness towards new ideas [41].

According to the bidirectional effect, foods that people consume affect their emotions and vice versa. Most of the choices associated with eating behavior are unarticulated and occur without insight or awareness. Thus, facial expression measurements reflect the dynamic sequence of emotional responses, whereas non-verbal reports correspond to the emotional status at the end of assessment [42].

There is no comprehensive sensory and consumer research in the literature [2,41,43,44,45,46,47,48,49] on the molecular and Note by Note meals in both cognitive impression and hedonic terms, which requires a special methodological approach.

It is worth determining the relationship between the expected and the experienced-liking of food products. The sensory expectations are associated with expectations of sensory descriptors such as sweetness and creaminess (at certain intensity), while the hedonic expectations are related to the extent the consumer likes or dislikes the food products [50]. According to Piqueras-Fiszman and Spence [51], the consumer’s expectations and perceptions may match or diverge; i.e., their expectations may be confirmed or disconfirmed. This phenomenon likely depends on the consumers and their attitudes, beliefs, personality and product familiarity. Many authors describe new, modernist dishes as a phenomenon in the restaurant business but have not studied sensory quality and emotions associated with it.

The aims of the study were (1) to determine the sensory characteristics of traditional, molecular, and Note by Note dishes; (2) to evaluate liking of key attributes (including expected- and experienced-liking) by consumers and their declared perceptibility of features and emotions in relation to dishes and (3) to observe the type and level of emotions and facial expressions of consumers in response to consumed dishes. As modernist cuisine is a relatively new alternative form of meals, food neophobia and innovativeness level of consumers have been determined. Willingness to try them in the future was also determined.

## 2. Materials and Methods

### 2.1. Material

A total of 6 dishes were prepared using lemon or tomatoes made in the traditional, molecular and Note by Note versions (Table 1). As a reference point, two traditional dishes were selected: tomato soup with rice and lemon butter cookies. The molecular version of the conventional dishes was prepared by modifying their texture, shape and/or temperature. The NbN version was designed by replacing conventional ingredients with as many technologically possible chemical compounds, distillates and NbN evocations (IQEMUSU SAS, France). A short ingredients list and description of the dishes’ preparation are presented in Appendix A (Table A1).

**Figure 1:**
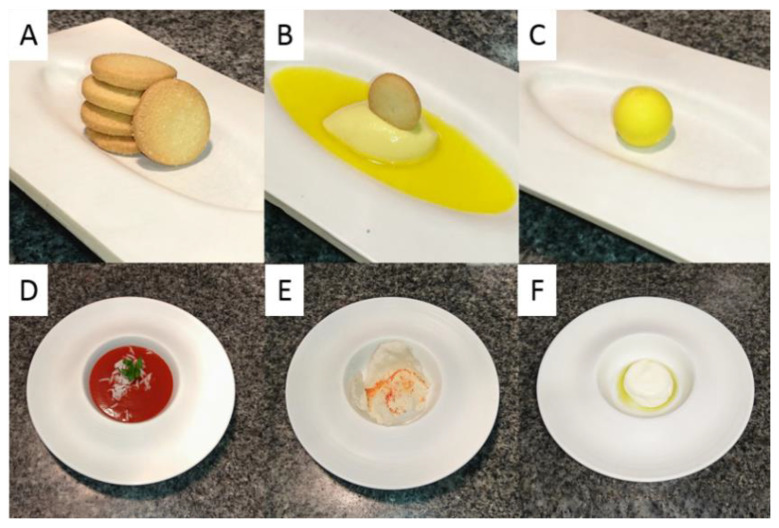
Plating proposition of: (**A**) lemon cookie, (**B**) molecular “lemon cookie”, (**C**) Note by Note “lemon cookie”, (**D**) traditional tomato soup, (**E**) molecular “tomato soup’, (**F**) Note by Note ‘tomato soup”.

The idea was to obtain completely new dishes that capture the quintessence of classic meals. The dishes (Figure 1) were designed and prepared by a professional chef who has worked in a Michelin-starred restaurant. The plan of the research was to evaluate the new version of foods that were visually different but in congruence with color and taste/flavor of classic products (lemon cakes) alongside dishes that were not in congruence in color (for example of tomato soup). Typical gastronomy equipment, and also Thermomix^®^VORVERK (Vorwerk Poland, Wrocław), Pacojet^®^2 (Zug, Switzerland) for ultra-comminution, and Centrifuge 5804 R (Eppendorf, Hamburg, Germany) for centrifugation were used for the preparation of molecular and NbN versions of dishes. The prepared products were complex and involved the presence of a sous-chef during all measurements. The dishes were not suitable for storage, so the chef prepared them immediately before the assessment.

### 2.2. Experiment Design and Methods

The study design involved two types of research:

1. sensory analytical tests,
2. consumer tests.

In the sensory analytical test, profiling was performed by trained panelists to provide objective data about products. The consumer tests included a survey aimed to characterize the tested group, as well as hedonic scaling and CATA to explore, respectively, liking of some attributes and declared perception of sensory features and emotions by consumers toward dishes. To investigate the mimic expressions assessment of consumers, the FaceReader was used.

#### 2.2.1. Sensory Analytical Method

*Sensory profiling*. The assessment was carried out using a Quantitative Descriptive Analysis. Sensory descriptors were selected and defined in accordance with the procedure of ISO 13299:2016 [52]. As a result, 16 of them were chosen for lemon-based dishes and 18 for tomato ones. The intensity of sensory attributes was assessed on an unstructured linear graphic scale (100 mm long), with specific word anchors on the edges (none on the left to very intensive on the right). The evaluation was conducted in three repetitions by a group of 8 panelists, who held expert qualifications [53] and have extensive experience in sensory analysis of innovative products.

#### 2.2.2. Consumer Study

Dishes testing included a survey and hedonic assessment performed by consumers (hedonic rating, CATA, FaceReader). Participants were informed that they would assess traditional dishes and their modernist versions.

*Questionnaire* was validated in a group of 4 people and consisted of 9 questions on their familiarity with the molecular and Note by Note cuisines’ terms and the frequency of their consumption. To estimate food neophobia level, the Food Neophobia Scale (FNS) by Pilner and Hobden [54] was used, while consumer innovativeness level was measured using the Domain-Specific Innovativeness (DSI) by Goldsmith and Hofacker [55] with modifications of Huotilainen et al. [56]. Ten statements applied from FNS and six from DSI were assessed on a 7-point scale and ranged from 1 = “strongly disagree” to 7 = “strongly agree” (Appendix A, Table A2). Consumers were also tasked with determining their willingness to try similar dishes in the future.

*Hedonic scaling.* A 9-point hedonic scale with expressions ranging from “extremely dislike” to “extremely like” [57] was used to quantify the degree of appearance, odor, taste/flavor, consistency and overall-liking (experienced). Additionally, prior to sensory testing, consumers were asked to observe samples and indicate their expected overall-liking of dishes based on their associations related to taste, flavor, and texture of presented dishes. The answer alternatives ranged from “extremely dislike” to “extremely like”.

*Check-All-That-Apply (CATA)*. The multiple-choice method was used to evaluate sensory-hedonic sensations and emotions associated with the dish consumption. Consumers had a choice of 22 different emotional attributes (surprising, common, intriguing, typical, disappointing, traditional, dietetic, artificial, delicate, rich, unique, flat flavor, atypical, tasty, interesting, innovative, trendy, boring, fresh, natural, aesthetic and processed).

In our study, attributes were selected by the assessors with a panel leader (after the profiling sessions of the dishes) and on the basis of a literature review made by the authors that was related to for example the use of CATA questions for product evaluation [58,59]. Knowing the sensory characteristics of the dishes was important in proposing the attributes to be identified. The final list of terms was developed by taking into consideration the (1) specificity of the assessed dishes, (2) panel discussion basing on the literature review made by the authors, (3) clarity of meaning terms for consumers (after translation into national language). The coordinator of the consumer assessment explained the principle of CATA questions prior to the evaluation of dishes [60]. Consumers had time to familiarize themselves with the selected attributes and ask questions. No problems with understanding the attributes were reported by consumers.

*Mimic expressions assessment.* The FaceReader 6 analyzing software (Noldus Information Technology, Wageningen, The Netherlands), connected to a web camera facing participants (angle < 40°), was used to assess mimic expressions in relation to the presented and tested dishes. The program, based on 491 model points, allows the recognition and real-time recording of seven facial reaction patterns: happy, sad, angry, surprised, scared, disgusted and neutral. A more detailed description of the software was published in a previous article [61,62]. The intensity of measured emotion was presented on a numerical scale, wherein 0 means not presented at all and 1 means the maximum value of the fitted model. The procedure for evaluation of the dishes was explained to participants before measurement began. In this study, due to various product textures and moments of swallowing, the emotions of subjects were recorded after the sample was placed in the mouth and for the next 50 s.

#### 2.2.3. Characteristics of Consumers

*The consumer testing* (CATA, hedonic scaling) was conducted in a group of 56 students and academics, who had reported any food allergies. Most were women (88.4%) and under the age of 25 (80.3%) Table 2. The FaceReader mimic expression was evaluated by 15 participants who declared a good mood and agreed to take part in the test.

FNS and DSI scales were used to sort respondents into appropriate groups. The range of possible FNS scores was from 10 to 70 c.u., wherein higher values corresponded to a greater neophobia level. The actual scores ranged from 12 to 50. The respondents were divided into three groups: the most neophilic (10.0–18.3), the most neutral (18.4–33.8) and the most neophobic subjects (33.9–70.0), in which the cutoff points were calculated by adding or subtracting one standard deviation (7.6) from the mean value (25.9). This classification has been applied in many studies, as reported by Vidigal et al. [32]. DSI scores could range between 6 and 42, but the actual scores were between 10 and 42. The mean value in the group was 26.3 (SD ± 5.6). Based on 33rd and 66th percentile points as cutoffs, consumers were classified into three groups: adapters (6–23), neutrals (24–28) and innovators (29–42).

More than half of the group (65.2%) was classified as the most neutral, while 20.5% of subjects were regarded as the most neophilic, that is, eager to try new products (Table 2). This is also supported by a higher percentage of innovators (34.8%) when compared with adapters (27.7%).

The majority (90.2%) of participants were familiar with the molecular cuisine term, but a smaller percentage (only 33.9%) had tasted it before (Table 2). The concept of the Note by Note cuisine is relatively recent, hence, not many consumers (26.8%) participating in the survey had encountered it before.

#### 2.2.4. Sample Presentation

Samples of the food (Table 1) were prepared and served immediately. Due to their different textures and consistencies, each was served at the temperature at which it would normally be consumed. They were given as a whole dish for sensory evaluation. Tomato soup and rice soufflé were served at 65 °C; lemon sorbet at −10 °C; cookie sphere and lemon consommé at 5 °C; lemon cookie and puffy rice snack at room temperature. The samples in appropriate quantities (20 g, the only exception was puffy rice snack, which weighed 10 g), were placed in plastic, transparent containers (100 mL) and then covered with a lid, marked with a 3-digit code and given to the assessors in a random order using the sequential monadic test [63]. Natural water was provided as a taste neutralizer between products.

#### 2.2.5. Testing Condition

Both sensory profiling and consumer study were carried out in an accredited sensory laboratory (contract No AB 564) that meets the requirements of ISO 8589:2007 [64]. The products were assessed in the same lighting and conditions to ensure focus on the assessor’s perception of sensory characteristics, emotions and liking.

To perform sensory profiling of the products and collect data, the computerized system ANALSENS was used. Panelists that had been trained in sensory analysis were used for profiling in two sessions per day, with sufficient relaxation time (3 h) interval between them. The assessors taking part in the test had about 15 years of experience in profiling of various products, including dishes.

Consumers evaluated the samples during a session in the afternoon of an assessment day that lasted approximately 25 min.

*Mimic expressions* of participants in relation to each food were observed during two sessions that lasted 8 min each and were separated by relaxation time of about 12 min. Lemon-based meals were followed by tomato-based meals.

### 2.3. Data Analysis

Statistical analyses were performed using XLStat 2017 (Addinsoft, Paris, France). The Shapiro-Wilk test was used to verify the normality of data distribution. The results were considered to be statistically significant at the level of materiality equal to 0.05.

Analysis of variance (ANOVA) with Fisher’s Least Significant Difference (LSD) post hoc test was performed to examine the differences in the intensity of attributes between tomato and lemon-based meals (traditional, molecular, NbN) considering products, assessors, and their interactions as fixed variables (the model two-way ANOVA with interactions).

Pearson correlation coefficients were calculated to relate the liking of given sensory characteristics and results of hedonic scaling of facial expression measurements in consumer tests.

The Kruskal–Wallis test was performed to analyze the differences in degree of liking of examined dishes. The relationships between degree of expected/experienced-liking and liking of particular sensory traits were analyzed by Spearman’s rank correlation.

Frequency of use of each one of the terms of the CATA question was determined by counting the number of consumers using a particular term to describe each product. To compare the type of dishes on CATA, results of the correspondence analysis were used. Cochran’s Q test was applied to CATA counts to determine whether there was significant difference in consumer perception for a given attribute among the examined dishes in terms of their preparation process. If significant differences were found among the variables, post hoc multiple pairwise comparisons were carried out using McNemar’s test with Bonferroni alpha adjustment. Correspondence analysis, based on chi-square distance, was performed to visualize associations between the CATA attributes and the tested products.

To determine the intensity of mimic expressions (FaceReader) in consumption time, the facial expressions of subjects were recorded after the sample was placed in the mouth and for the next 50 s. Then, from 750 records (individual face shots) per person consisting of 7 numbers quantifying emotions, 11 records corresponding to every 5 s of measurement were extracted, and the average for the group was calculated. To determine the intensity of the expressions for swallowing-related time, the moment of swallowing for individuals was determined using video recordings; then, 6 records representing −10, −5, 0, +5, +10, +15 in relation to swallowing were extracted, and the average for the group was computed.

## 3. Results

### 3.1. Sensory Characteristics of Different Dishes

The modern dishes may differ in sensory properties from traditional counterparts despite similar general assumptions. According to the “food deconstruction” technique, a new dish should preserve the essence of classic one (in our case: tomato soup—tomatoes, rice, roasted meat; lemon cookie—lemon, cookie dough). It becomes essential to recognize the qualitative and quantitative sensory dimensions of the examined dishes.

**Figure 2:**
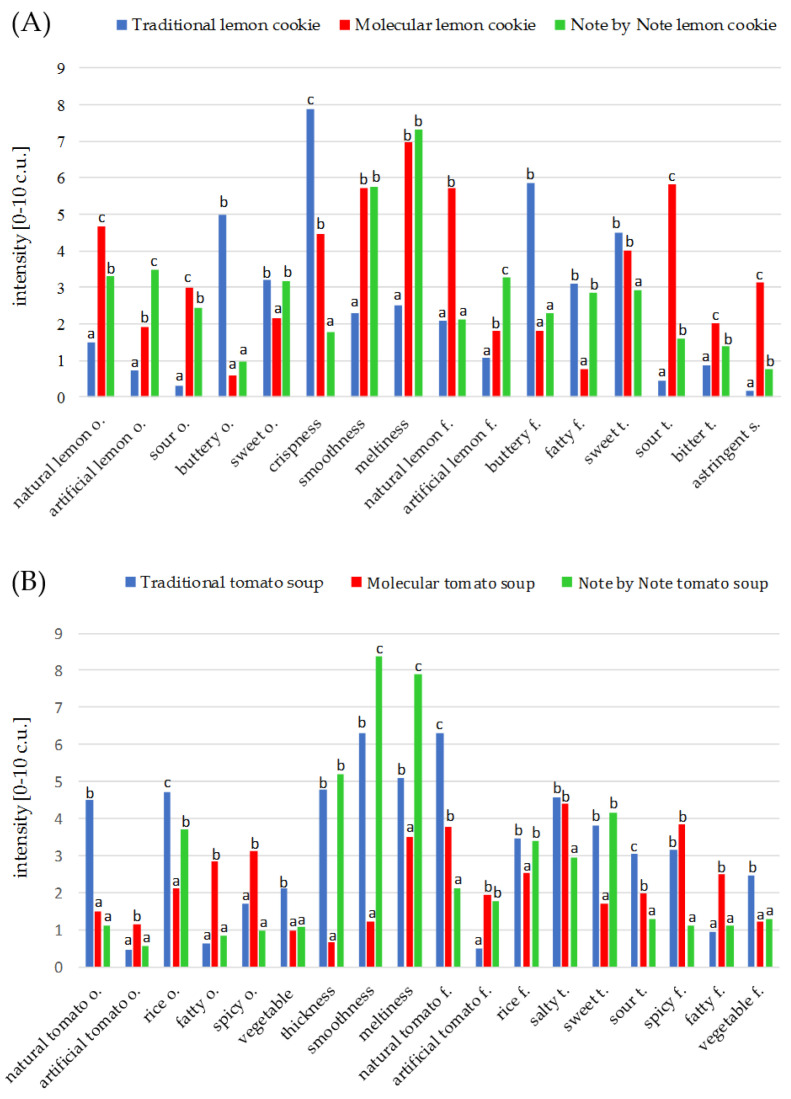
Sensory properties of (**A**) lemon cookie and (**B**) tomato soup that are prepared in traditional, molecular and Note by Note versions: a, b, c—mean values marked by different letters in versions of dishes differ significantly at *p* ≤ 0.05.

Sensory characteristics depended mainly on the type of dishes (traditional, molecular or Note by Note) for both lemon cookies and tomato soups (Figure 2A,B). Traditional versions of the dishes, regardless of their type, highlighted the characteristic (typical) attributes. For example, the intense butter note and crispy texture distinguished traditional cookies from others. Among different versions of tomato soup, the highest intensity of typical natural tomato odor and flavor, vegetable note and sour taste were found in traditional products.

In contrast, modern cuisine caused a statistical increase or decrease in the intensity of some key attributes that are related to odor, flavor, taste and texture. The molecular and NbN versions of cookies showed a greater level of smoothness and meltiness than the traditional ones. The intensity of many odors and taste/flavor attributes were also different. Molecular cookies were characterized by the highest level of natural lemon odor and flavor, sour taste, and astringency, while the Note by Note cookies revealed the most artificial lemon note (Figure 2A). In turn, the lowest sensation of rice odor and flavor and of sweet taste and the greatest fatty and spicy odor and flavor were observed in molecular tomato soup (Figure 2B). In terms of consistency, traditional and NbN soups revealed a very similar level of thickness, but the NbN sample had the greatest smoothness and meltiness. The molecular version had a completely different texture.

### 3.2. Consumer Liking of Innovative Food

The evaluation of dishes is a complex experience that includes sensory, emotional and ideational components. For this reason, the experienced and expected-liking and the influence of factors associated with consumer attitudes toward new food were explored.

**Figure 3:**
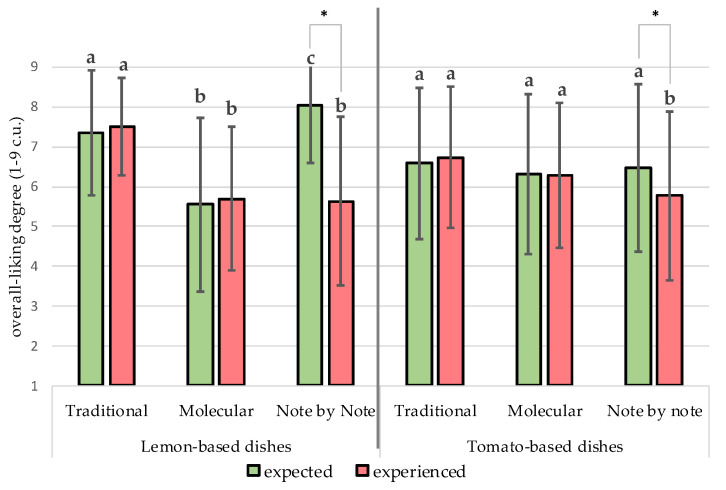
Expected and experienced overall liking in relation to dishes: a, b, c—values of expected-liking or experienced-liking that are marked by different lowercase letters between various versions of dishes with the same main ingredients differ significantly at *p* ≤ 0.05; * asterisk brackets indicate a significant difference between expected and experienced overall liking of the given sample at *p* ≤ 0.05.

*Hedonic Image.* Expected and experienced-liking of traditional and molecular versions of lemon- and tomato-based dishes were not varied (Figure 3). However, the expected-liking of NbN in both meals was significantly higher than the experienced-liking.

The highest positive correlations were observed between the expected-liking and appearance-liking (rho = [0.33–0.73]; ***p*** ≤ 0.05). In turn, the experienced-liking depended significantly on flavor-liking (rho = [0.82–0.88]; *p* ≤ 0.05) and texture-liking (rho = [0.61–0.80]; *p* ≤ 0.05). Generally, the liking based on appearance of NbN dishes had the lowest effect on experienced liking.

**Figure 4:**
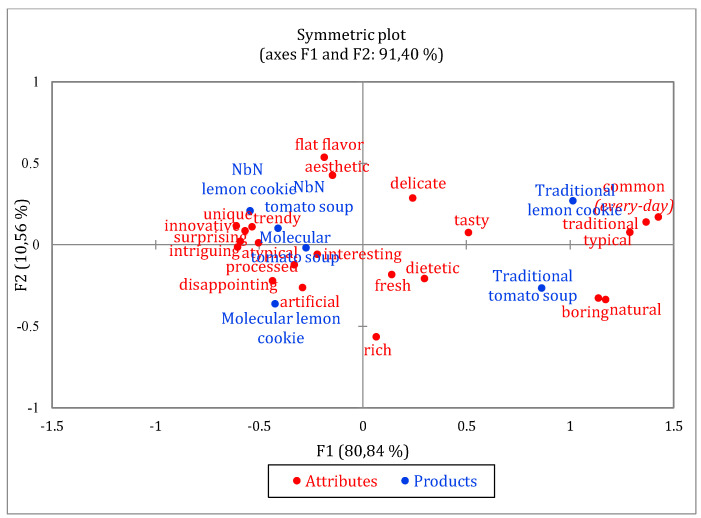
The dishes and term representation in the first and second coordinates of the Correspondence Analysis performed on the frequency of use of attributes by consumers (according to check-all-that-apply).

All the dishes received moderately high hedonic scores; however, their deeper examination showed differences in the degree of liking, depending on type of dish and their version. The hedonic scores for the experienced-liking of the Note by Note dishes were lower than for traditional versions (*p* ≤ 0.05) but did not vary from scores of molecular courses (Figure 4). The odor, taste/flavor, and texture-liking for traditional meals were significantly higher (*p* ≤ 0.05) than molecular and NbN versions of lemon-based meals and the NbN version of tomato meals (Table 3).

*Factors influencing liking.* The higher standard error of the mean hedonic scores exhibits varied affective response of consumers (Table 3, Figure 3). The effect of food neophobia and consumer innovativeness’ levels were evaluated. The explanation of the results might be found in the Food Neophobia Level (Table 2). Higher levels of Food Neophobia (FNL) of evaluators resulted in less liking for texture (r = −0.27, *p* ≤ 0.05) for the molecular cookie. Moreover, the higher FNL, the lower the experienced-liking for the NbN cookie (r = −37, *p* ≤ 0.05). This phenomenon was especially evident in the group of the most neophobic participants. The difference between mean values of expected and experienced-liking was the highest (2.0–4.7 c.u.) in the group of the most neophobic subjects, while in the other groups (the most neutral and the most neophilic participants), it ranged between 0.4–2.2 c.u.

As consumer innovativeness level increased, the flavor-liking (r = −0.36, *p* ≤ 0.05) and experienced-liking (r = −0.40, *p* ≤ 0.05) of traditional lemon cookie decreased. The familiarity with novel cuisines did not show any significant effect on the liking.

*Willingness to try by consumers innovative foods in the future.* A higher percentage of consumers showed a willingness to try similar traditional dishes (71.9–86.0%) in the future, than the molecular (54.4–61.4%) or Note by Note (59.6–64.9%) ones (not shown in tables). However, the percentage of people who did not want to try molecular (17.5–31.6%) or NbN (17.5–19.3%) versions was similar to that of the undecided consumers (15.8–21.1% and 17.5–22.8%, respectively). The mean values of liking in a group of consumers who would not eat modernist dishes again were lower than 4.5 c.u. The mean score of undecided consumers (5.1–6.5 c.u.) was lower than those who declared a willingness to eat again (6.5–6.8 c.u.). This may indicate that the consumption of dishes prepared with these innovative techniques introduces a dissonance between unfulfilled sensory expectations and the desire to be surprised.

### 3.3. Emotions and Sensory-Hedonic Sensations of Meals

The sum of word expressions indicated by consumers used for the survey (*n* = 56) revealed (data not presented) that molecular and Note by Note dishes evoked more emotions than traditional versions. The NbN cuisine aroused the most emotions (366–405 indications in total), followed by molecular cuisine (300–330) and the least traditional cuisine (303–308).

Data analysis further showed that traditional cuisine compared to modernist ones were associated with word expressions such as tasty (43 indications per meal), traditional (35) and typical (31). The proportions of selections by consumers for each attribute of CATA question for all dishes was presented in a contingency table (Table 4).

A higher proportion, e.g., closer to 1.00, indicates that, among the six dishes, the attribute was more frequently chosen by consumers. Cochran’s Q test revealed differences for 19 of the 22 terms of the CATA questions used to characterize the dishes. The dishes did not vary in attributes such as dietetic, artificial, and fresh. As the use of processed ingredients increased, the number of the following indications increased: surprising, intriguing, innovative, atypical, unique and trendy. These relationships can be clearly seen on a bi-plot of correspondence analysis (explaining 91.4% of total variance), which show associations between the type of dishes (culinary techniques) and emotional descriptors or hedonic sensations. Along with the level of food processing, e.g., for lemon-based dishes (Classic → Molecular, Note by Note cuisine), the disappointment increased when the naturalness sensation decreased (Figure 4). Interestingly, a larger share of consumers perceived NbN dishes as slightly more unique, trendy and innovative than molecular ones.

### 3.4. Mimic Expressions Assessment: Type and Level of Emotions in Relation to Dishes in Time

Consumption of traditional meals aroused fewer facial expressions among consumers than molecular or Note by Note ones. In the case of traditional courses, the intensity of neutral facial expression ranged between 0.38 and 0.58 c.u., while in molecular and NbN dishes were lower (respectively, 0.28–0.53 and 0.26–0.53 c.u.). The level of neutrality depended on the moment of consumption. It was at a stable level in traditional dishes, while in the case of modernist cuisines, it had different dynamics in time. The consumption of molecular and Note by Note dishes evoked fewer neutral facial expressions while chewing, which rose significantly after swallowing samples. Neutrality rose 4.9–9.2% in traditional versions, 16.6-24.5% in molecular cuisine and 29.1–33.4% in NbN.

Mean values from facial expression measurement (*n* = 15) and mean values of experienced-liking (*n* = 56) revealed a significant positive correlation (*p* ≤ 0.05). Higher intensity of neutrality (only during the first 10s of measurement) and lower intensity of disgust in 15s of assessment (average deglutition time) resulted in higher scores for experienced-liking (respectively r= 0.82–0.92 and r = 0.96).

**Figure 5:**
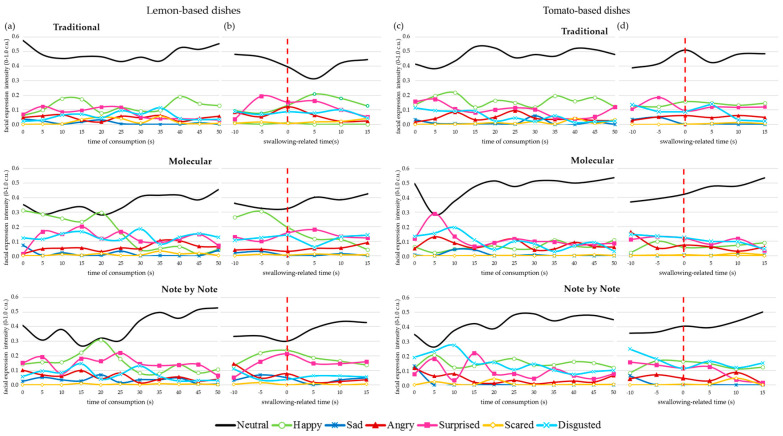
Real-time mimic expresions intensity towards dishes based on (**a**,**b**) lemon and (**c**,**d**) tomato: (**a**)—during entire consumption time of lemon-based dishes; (**b**)—for the swallowing-related time of lemon-based dishes; (**c**)—during entire consumption time of tomato-based dishes; (**d**)—for the swallowing-related time of tomato-based dishes.

Analysis of facial expressions during the entire consumption time revealed that the dominant expression (excluding neutrality) associated with the eating of traditional lemon cookie and tomato soup was happiness (Figure 5a,c). Although kept at a relatively constant level, the emotion was more intense than other emotions from 5 to 20 s after placing the sample in the mouth and from 35 s until the end of measurement.

In the case of molecular and NbN versions, quite different dynamic changes in expressions can be observed. Moreover, facial expression status during consumption depended on the type of dish. The common feature for both of the lemon-based products examined is that the highest peaks of happiness appear around 20 s from placing the sample in the mouth and then disappear (Figure 5a). Simultaneously, moderately high intensities of surprise mixed with disgust are exhibited during the entire consumption.

Consumers reacted differently for the tomato puffy rice snack. Its consumption did not evoke any happiness, and during the first 10 s of FaceReader measurement, a high level of surprise rose and then decreased (Figure 5c). After 15 s of measurement, the emotions decreased, probably as the result of snack texture. Mimic expressions assessment of the rice soufflé with tomato gel revealed that the dominant expression prior to 15 s of measurement was disgust, followed by happiness. Afterwards, notable peaks of surprise and happiness were recorded. Some facial mimic expression may be related to the sensory properties of dishes (Figure 3) and the feeling of aftertaste in time, both positive and negative.

The analysis of facial expressions dynamics for swallowing-related time (Figure 5b,d) revealed that prior to the swallowing (−5 s) of both traditional dishes, slight surprise peaks were noticed, followed by a neutrality decrease (+5 s). In the case of molecular and NbN lemon cookies, notable (especially in the molecular version) peaks of happiness were noticed 5 s before swallowing. At 5 s before the swallowing of molecular and NbN versions of tomato soup, a slight facial expression of disgust was noticed. In all molecular and NbN versions, a growing trend of neutrality was observed, which was opposite from classical ones.

## 4. Discussion

The detailed sensory profile is a very important tool for new product development. It allows one to explore the effects of ingredients and processing variables on the final sensory quality of foods and dishes. Moreover, such information is very useful for understanding consumer responses in relation to products’ acceptance. The present study revealed that each version of the lemon and tomato dishes (traditional, molecular, NbN) had a separate sensory pattern (profile) related to technological aspects of their production and the recipe used. It is crucial for cooks and producers to maintain a balanced intensity of attributes in various meals that determine their identity and specificity regardless of the modernization process. Thus, too intense or weak intensity of, for example, key flavor descriptors and basic tastes like sourness or sweetness can result in a hedonic response in relation to a product. That, in turn, can potentially affect the degree of liking by consumers and their positive or negative experiences, including evoked emotions [65]. According to Spinelli and Jaeger [66], the sensory characteristics of a product may be altered in order to increase specific positive emotions or even to decrease negative emotions among consumers. This seems crucial in modern cuisine and it is also intriguing due to the atypicality of the dishes.

Visual appeal of the product is an important driver of sensory expectations, which was confirmed in the present study by the highest correlation of expected-liking and appearance-liking. The experienced-liking of NbN dishes was lower than that of traditional ones. The most important factor for consumers was the flavor/taste of the product. These results are in accordance with findings of Santagiuliana et al. [67], who stated that the visual appearance of novel heterogeneous foods can significantly affect expected-liking but not the actual-liking. The experienced-liking is affected by textural and flavor oral sensory perceptions. However, visual cues can significantly influence consumer texture perception. Moreover, literature [68] confirmed that consumers primarily pay attention to flavor-liking when evaluating the overall-liking of products. The examined meals, depending on the type, had a completely different appearance, which could determine the perception of taste and flavor. It is intriguing the extent to which the consonance of the color with the product (yellow lemon cookies) affected the sensory perception of consumers in contrast to the incongruent color of dishes in the case of tomato soup (e.g., white NbN version). Moreover, different textures of the dishes could affect the results of the assessment. Liking of evaluated dishes may be related to their sensory profile. For example, low intensity of buttery attributes as well as a higher level of lemon note in the molecular cookie and artificial lemon attributes (odor and flavor) in NbN compared to the traditional products could determine a lower hedonic response of consumers. Similarly, the significantly lower intensity of tomato flavor and aroma in modern cuisine (molecular, NbN) probably affected the lower results of liking compared to the traditional option. According to other studies [69,70], change of texture and appearance of familiar food in novel food development are only possible when similar retro-nasal sensation is ensured. Consumers are sensitive to the modification of the expected texture. Traynor et al. [48] emphasize that besides intrinsic sensory properties, extrinsic motivation like emotional reactions have a significant effect on the acceptance of novel foods.

Consumer expectations regarding odor, taste, and texture-liking of modernized versions of dishes were probably higher. Similar results were demonstrated in a study by Stolzenbach et al. [71], where the mean scores for liking were significantly higher for the traditional honey than for the novel kind. Moderately high sensory ratings of the molecular version of Portuguese custard tart ‘Pastel de Nata’ were stated by Oliveira et al. [72]. The lowest appearance-liking for the molecular version of lemon cookies may be the result of food looking more chaotic (cookie chunk stuck into sherbet, which is served in soup). On the other hand, a congruent color with a product representing an unusual shape influenced a significantly higher expected overall-liking when compared to the tomato version (white soufflé soup color, NbN version). This is in line with the research of other authors. Zellner et al. [73] and Zellner et al. [74] provided scientific evidence that foods presented in a less neat manner are less liked. Similar to our findings, results of studies with novel salad dressings [75,76] and with other novel foods [77] indicated a higher mean of expected acceptability in a group of neophiliacs than neophobics.

According to the psychological theories, there are differences in consumer’s product expectations [51]. Piqueras-Fiszman and Spence [51] stated that disconfirmation (to any degree) between a consumer’s product expectations and the experience can be explained by the two phenomena. The assimilation is one of them, and it happens when the consumer tries to minimize differences between the perception of the product and its expectation, while the contrast happens when the consumer tries to maximize these differences. In our study, consumer attitudes toward examined meals include these two aspects. The assimilation probably occurred for the first two methods (traditional and molecular meals), while the contrast appeared for NbN. This suggests that expectations of consumers were set too high, which resulted in a certain level of dissatisfaction. This also may indicate that the consumption of dishes prepared with these innovative techniques creates dissonance between unfulfilled sensory expectations and the desire to be surprised by novelties. It is worth mentioning that small deviations from the level of consumer adaptation can be seen as interesting and novel. Large deviations tend to result in disgust or neophobia instead [51,54]. It is worth emphasizing that the research results could be somewhat different if the meals were assessed in a different surrounding, for example, in a restaurant. According to many authors [31,44,78] consumers may be more willing to tolerate or expect a certain level of incongruency in some contexts (e.g., modernist/experimental restaurants).

CATA questions provided more profound insight and better explain consumers’ perceptions and liking of modern cuisines (molecular and NbN versions of dishes). Traditional cuisine compared to modernist ones were associated with being tasty, traditional, and typical. In contrast, novel cuisines we more frequently perceived as surprising, intriguing and innovative. Our results appear to be well substantiated by the results of several authors, who compared traditional foods with their novel versions. Consumers perceived novel beers as “unusual”, “complex”, “intriguing” and for “special occasions” [79]; molecular dishes as “surprising”, “arousing curiosity”, “innovative” and “liked” [41]. Moreover, a familiar type of chocolate was associated with positive emotion terms (e.g., sweet—"happy”), but new chocolates (e.g., with salt) evoked various emotions, including “bored”, “interested”, or “a little naughty” [80]. Loss et al. [81] also concluded that the acceptance of very innovative foods go beyond neophobia. The results of previous studies [81,82] reveal that traditional versions of selected foods were more liked in comparison to their modernized versions. Additionally, the most unusual foods, arousing curiosity and surprise and challenging for senses, were least liked. Acceptance of unfamiliar food is mainly dependent on its degree of similarity to familiar food [44], which aligns to some extent with the results of our research. In this context, it should be emphasized that the unique, aesthetic and atypical appearance of new products should also correspond with the experienced and balanced taste and flavor that provide satisfaction, evoke positive emotions and affect the degree of liking. It seems that this will be met in particular when the expected-liking is relatively high, but equal to the experienced-liking or even lower. In a previous study [41], modernist cuisine also aroused more positive emotions (35.3%) in consumers than the traditional one.

Mimic expressions assessment by FaceReader was a measure to capture the type and level of emotions as temporal effects in relation to examined meals. According to Danner and Duerrschmid [83], the unfolding of emotions can provide deeper insight into the evaluation process itself and the formation of liking. Our study revealed that higher neutrality during 10 s and disgust in 15 s of consumption was related with higher experienced-liking. These findings align with the result of Danner et al. [84], who found a high positive correlation between ‘‘neutral’’ facial expression with liking, as well as a high negative correlation of ‘‘angry’’ and ‘‘disgusted’’ facial expression with liking. Neutral emotion is frequently used to describe hedonically liked stimuli [85]. In He et al. [42] study, pleasant odors were related to more intense neutral and surprised facial expressions as well as less intense disgust emotion. On the other hand, unpleasant odors evoked fewer neutral reactions and a more intensified disgust. Generally, a higher concentration of odors resulted in lower facial neutrality and more scared expressions. It should be noted that fewer consumers (*n* = 15) agreed and participated in the FaceReader test than in other tests performed with consumers. According to Crist et al. [86], a range of 10–50 participants would take part in the study using automated facial expression analysis software. They found that the number of participants would vary depending on flavors, flavors intensity and expected treatment acceptability. It was emphasized that products with smaller flavor differences require more participants. Our dishes varied significantly in sensory characteristics (profiling data). Nevertheless, the results of the research with FaceReader should be considered as preliminary. 

More consumers would like to eat similar traditional dishes in the future than molecular or NbN dishes. Consumers were a little disappointed by NbN. This was a result of the unfamiliar appearance of dishes, especially the tomato-based dish. The lemon-based dish in NbN version was similar to a dessert, and it affected the evaluation. According to Burke et al. [87], making the NbN dish that looks familiar to the consumers may help to overcome their neophobic reaction. The inspiration from traditional foods may contribute to the success of NbN cooking and cuisine. In previous studies, consumers declared moderately high willingness to buy the molecular vinaigrette jellies (42%), a port wine faux caviar (58%) or powdered olive oil with flavors (72%) [2,45,46]. Most of the participants (88%) in our recent study [41] would like to taste different molecular courses in the future. In turn, consumers in the study by Mielby and Frøst [44] did not want to eat the most innovative and highly unusual molecular dishes again. This is supported by the research of Oliveira et al. [72], wherein purchase intention was moderately high. Tan et al. [88] have also reported that willingness to eat novel burgers with insect additions was lower than regular beef patties. Their findings suggest that consumers may be inclined to taste novel (unusual) foods to satisfy their curiosity, but not to consume them again, especially in the case of low sensory-liking or inappropriateness for consumption. The results of Barenna and Sánchez’s [36] study suggest that decisions to consume novel foods have a more pronounced emotional component in a group of neophobic consumers. Hence, neophiliacs try novel foods in restaurants more often than the neophobes [89]. Neophiliacs had a higher awareness, willingness to try and rated unfamiliar food more favorably [75].

The detailed sensory profile is a very important tool for new product development. It allows us to explore the effects of ingredients and processing variables on the final sensory quality of foods and dishes. Moreover, such information is very useful for understanding consumer responses to modernist products such as molecular and NbN dishes. Higher results of expected-liking compared to Note by Note experienced-liking showed disappointment with this version which may be related to the flavor and taste of the dishes. Additionally, significantly lower overall liking of NbN dishes compared to classic ones suggests that the success of novel dishes might be related to the other elements of multisensory experience. This type of modern cuisine is not designed for a large audience. It seems to be intended for small groups of consumers, with low neophobia and who are very familiar with modern cuisine. This finding requires further research.

## 5. Conclusions

Our findings show that the expected-liking of dishes depended on their type. The results may be related to appearance and especially color–taste congruency with the dishes, which affect consumer associations and expectations regarding sensory characteristics. Moderately high liking scores of molecular and Note by Note (NbN) cuisines combined with the structure of the emotional response suggests the new insight that consumption of such dishes is as result of temporary emotional arousal derived from an element of novelty. Consumption of modernist dishes evoked more mimic expressions among participants than traditional ones, especially during the first phase (chewing). This facial emotional arousal was confirmed by a number of declared emotions and hedonic sensations. The success of modernist dishes that are served at high-end restaurants is not only strongly associated with acceptable intrinsic sensory characteristics but also with enhanced emotional reactions.

On the other hand, it is worth emphasizing that food neophobia and consumer innovativeness had significant effects on the liking of given products and affected the attitude of consumers towards modern dishes and their willingness to try them again. A higher percentage of consumers showed a willingness to try similar traditional dishes in the future than molecular or NbN dishes, with more than half of the consumers indicating they would eat similar modernist dishes. This may indicate that the consumption of dishes prepared with these innovative techniques introduces a dissonance between unfulfilled sensory expectations and the desire to be surprised by novelties.

Traditional cuisine was associated with a greater number of consumers’ word expressions such as tasty, traditional and typical compared to modern ones. A higher level of food processing (molecular cuisine → Note by Note cuisine), caused more participants to perceive meals as surprising, intriguing/interesting and innovative. The uniqueness of modern dishes was most related to innovativeness and being surprising and intriguing, while disappointment in modernist dishes may have been associated with a lower sensation of naturalness (e.g., lemon-based dishes) compared to classical ones.

New food product developers should take into account the attitudes and expectations of potential consumers. In the future, focus groups could be planned to identify different consumer expectations for new products. Such an approach would be valuable in modifying features like the taste, flavor and texture of dishes according to consumers’ points of view. Culinary workshops for consumers combined with the preparation and evaluation of such kinds of dishes would be also interesting.

Learning about the perception of modernist cooking solutions enables a better understanding of the sensory experiences of potential consumers, which becomes the space and inspiration for innovation. According to these findings, and taking into account the fact that it is difficult to assess the type of customers trying the dishes (neophiliacs, neophobes, neutrals), we believe that more attention should be given to the appearance of new products. The acceptance of innovation is also linked to the risks of failure. If we change the appearance of the food too much, the exposure to failure will be increased.

## 6. Limitation

A limitation of our research is that assessments of hedonic and other tests were carried out under laboratory conditions, while the multisensory experience of modernist cuisine implies that it is associated with the restaurant atmosphere, including social contacts and service provided. Moreover, the evaluation of these innovative dishes was performed by comparison to traditional dishes, which could have had an impact on the results.

Application of the immersive approach would recognize contextual influences (e.g., external variables like colors, light, temperature) on perception of modernist dishes, simultaneously with consumer engagement. Such research would allow a deeper analysis and understanding of consumers behavior and expectations towards molecular cuisine and Note by Note cooking.

Another limitation of our research is the number of consumers taking part in the experiment. There were only 56 consumers that took part in the experiment. Amongst them, more than a half were classified as the most neutral, and about 20% were categorized as the most neophiliac, who are eager to try new products.

## 

## Figures and Tables

**Table 1:** The versions of dishes used in the study.

Main Ingredients	Version of Dish		
	Traditional (Classical)	Molecular	Note by Note
Tomato-based dishes (tomatoes, rice)	creamy tomato soup with rice technique: boiling	puffed rice snack with heirloom tomato and roasted meat powders techniques: powdering; rice snack: deep frying	rice soufflé with the roasted meat essence and tomato gel technique: foaming
Lemon-based dishes (lemon, butter)	lemon cookie technique: baking	butter cookie with lemon peel sherbet with and lemon consommé techniques: baking, freezing, evaporating	cookie-flavored sphere with a lemon coating techniques: freezing, coating, centrifugation

**Table 2:** Characteristic of participants (*n* = 56).

Characteristic		Percent of Consumers (%)
Gender	Woman	88.4
	Man	11.6
Age	<25	80.3
	25–40	12.5
	41–56	7.2
Food Neophobia Level *	The most neophilic (10.0–18.3)	20.5
	The most neutral (18.4–33.8)	65.2
	The most neophobic (33.9–70.0)	14.3
Domain-Specific Innovativeness level **	Adapters (6–23)	27.7
	Neutrals (24–28)	37.5
	Innovators (29–42)	34.8
Familiarity with the molecular cuisine term	Yes	90.2
	No	9.8
Possibility of earlier tasting of molecular cuisine	Yes	33.9
	No	66.1
Familiarity with the Note by Note cuisine term	Yes	26.8
	No	72.4
Application of ingredients based on synthetic substances at home (e.g., vanillin, bouillon cube)	Yes	84.8
	No	15.2

* Adapted Food Neophobia Scale (10–70 c.u.) [34]; ** Domain-Specific Innovativeness Scale (6–42 c.u.) (Goldsmith and Hofacker [55] with modifications of Huotilainen et al. [56]).

**Table 3:** Degree of liking of evaluated dishes.

Sensory Trait	Lemon-Based Dishes			Tomato-Based Dishes		
	Traditional	Molecular	NbN	Traditional	Molecular	NbN
	Degree of Liking, (1–9 c.u.) $\bar{x}$ ± SE; *n* = 56					
appearance	7.4 ± 0.2 ^b^	5.2 ± 0.3 ^a^	8.2 ± 0.2 ^c^	6.5 ± 0.3 ^a^	6.6 ± 0.3 ^a^	6.0 ± 0.3 ^a^
odor	8.0 ± 0.2 ^c^	6.3 ± 0.2 ^a^	6.9 ± 0.3 ^b^	6.8 ± 0.2 ^b^	5.4 ± 0.3 ^a^	6.0 ± 0.3 ^a^
taste/flavor	7.5 ± 0.2 ^b^	5.5 ± 0.3 ^a^	5.2 ± 0.3 ^a^	6.8 ± 0.3 ^b^	6.2 ± 0.3 ^ab^	5.5 ± 0.3 ^a^
texture	7.9 ± 0.2 ^b^	5.5 ± 0.3 ^a^	5.8 ± 0.3 ^a^	7.0 ± 0.2 ^b^	6.9 ± 0.3 ^ab^	6.2 ± 0.3 ^a^

^a, b, c^—mean values marked by different letters in verses, differ significantly at *p* ≤ 0.05.

**Table 4:** A contingency table of data set with 6 products and 22 emotional attributes of the CATA question.

Attributes	Lemon-Based Dishes			Tomato-Based Dishes			*p*-Value (Cochran’s Q)
	Traditional	Molecular	NbN	Traditional	Molecular	NbN	
surprising	0.018 ^a^	0.298 ^b^	0.412 ^b^	0.035 ^a^	0.316 ^b^	0.395 ^b^	0.000
common	0.237 ^b^	0.000 ^a^	0.000 ^a^	0.175 ^b^	0.018 ^a^	0.000 ^a^	0.000
intriguing	0.053 ^a^	0.281 ^b^	0.368 ^b^	0.053 ^a^	0.263 ^b^	0.333 ^b^	0.000
typical	0.289^b^	0.009 ^a^	0.000 ^a^	0.246 ^b^	0.035 ^a^	0.018 ^a^	0.000
disappointing	0.009 ^a^	0.140 ^b^	0.149 ^b^	0.053 ^ab^	0.061 ^ab^	0.079 ^ab^	0.000
traditional	0.342 ^b^	0.000 ^a^	0.000 ^a^	0.272 ^b^	0.026 ^a^	0.018 ^a^	0.000
dietetic	0.026 ^a^	0.009 ^a^	0.000 ^a^	0.070 ^a^	0.070 ^a^	0.053 ^a^	0.011
artificial	0.018 ^a^	0.132 ^a^	0.114 ^a^	0.079 ^a^	0.105 ^a^	0.096 ^a^	0.052
delicate	0.298 ^b^	0.061 ^a^	0.167 ^ab^	0.167 ^ab^	0.158 ^ab^	0.281 ^b^	0.000
rich	0.070 ^a^	0.281 ^b^	0.088 ^a^	0.263 ^b^	0.167 ^ab^	0.114 ^a^	0.000
unique	0.009 ^a^	0.096 ^ab^	0.184^b^	0.035 ^a^	0.140 ^b^	0.237 ^b^	0.000
flat flavor	0.035 ^ab^	0.000 ^a^	0.123 ^b^	0.026 ^ab^	0.035 ^ab^	0.026 ^ab^	0.000
atypical	0.000 ^a^	0.175 ^b^	0.246 ^b^	0.035 ^a^	0.088 ^ab^	0.211 ^b^	0.000
tasty	0.430 ^b^	0.114 ^a^	0.114 ^a^	0.325 ^b^	0.272 ^ab^	0.175 ^a^	0.000
interesting	0.132 ^a^	0.263 ^ab^	0.263 ^ab^	0.158 ^a^	0.289 ^ab^	0.333 ^b^	0.003
innovative	0.009 ^a^	0.149 ^b^	0.298 ^c^	0.018 ^a^	0.211 ^bc^	0.219 ^bc^	0.000
trendy	0.018 ^ab^	0.105 ^abc^	0.175 ^c^	0.009 ^a^	0.123 ^bc^	0.123 ^bc^	0.000
boring	0.053 ^ab^	0.009 ^ab^	0.009 ^ab^	0.096 ^b^	0.009 ^ab^	0.000 ^a^	0.000
fresh	0.132 ^a^	0.158 ^a^	0.079 ^a^	0.114 ^a^	0.088 ^a^	0.096 ^a^	0.379
natural	0.158 ^b^	0.026 ^a^	0.000 ^a^	0.272 ^b^	0.026 ^a^	0.018 ^a^	0.000
aesthetic	0.149 ^ab^	0.053 ^a^	0.263 ^b^	0.044 ^a^	0.132 ^ab^	0.149 ^ab^	0.000
processed	0.035 ^a^	0.123 ^ab^	0.123 ^ab^	0.053 ^a^	0.193 ^b^	0.096 ^ab^	0.001

Cochran’s Q test was carried out to determine whether the proportions of selection by consumers for each attribute of the CATA question differed, taking into account the assessed dishes. Post hoc multiple pairwise comparisons were carried out using McNemar’s test with Bonferroni alpha adjustment. Different superscript letters within each row denote significant differences (*p* ≤ 0.05).

## Appendix A

**Table A1:** The preparation procedures of dishes used in research.

Version of Dish	Name of Dish	Ingredients	The Preparation Procedures
Lemon-Based Dishes			
Traditional (classical)	lemon cookie	flour, egg yolks, milk, butter, sugar, baking powder and lemon zest	Traditional lemon biscuit. The dough was prepared by mixing cold butter with the rest of the ingredients. Next, the dough was rolled, molded and baked.
Molecular	butter cookie with lemon peel sherbet with and lemon consommé	flour, egg yolks, butter, sugar, lemon, glucose, stabilizer, citric acid, xanthan gum and heavy cream	Lemon sherbet was made in PACOJET by comminution of the frozen purée. The puree consisted of cooked lemon zests, lemon juice, sugar sirup, stabilizer, xanthan gum and heavy cream. Lemon consommé was made by reducing a mixture of glucose syrup and lemon juice in a rotary evaporator.
Note by Note	cookie-flavored sphere with a lemon coating	clarified cookie water, xanthan gum, cocoa butter, milk powder, NbN evocation (biscuit and lemon flavors), yellow food colorant (Tartrazine)	In this version, the butter biscuits were mixed with water. After maceration, the liquid was separated using centrifuge. It was then mixed with xanthan gum, milk powder and NbN biscuit evocation followed by freezing in a silicone spherical form. Once frozen, the spheres were then immersed in a coating. The coating was made with cocoa butter, yellow colorant and NbN lemon evocation.
Tomato-based dishes			
Traditional (classical)	creamy tomato soup with rice	chicken wings, milk powder, sunflower oil, carrot, celery, parsley, onion, fresh bay leaf, allspice, thyme, pelati tomatoes, onion, garlic, tomato paste, salt, pepper, basmati rice	The stock of roasted wings, vegetables and seasonings was pressure cooked and then reduced. Next tomato, onion, garlic, and tomato paste was added. All ingredients were subsequently blended in Thermomix^®^. Finally, the soup was served with cooked rice.
Molecular	puffed rice snack with heirloom tomato and roasted meat powders	basmati rice, sunflower oil, maltodextrin, dried heirloom tomatoes, pork sirloin, bay leaf, allspice, thyme, salt, pepper	A mix of heirloom tomato puree and maltodextrin and roasted pork meat (with seasoning) were dried, powdered and served on a puffed rice snack. The snack was made of overcooked rice blended with salt, spread thinly on the sheet pan, dehydrated and then deep-fried in sunflower oil.
Note by Note	rice soufflé with the roasted meat essence and tomato gel	basmati rice, whole milk, kuzu starch, xanthan gum, egg white, sucrose, dextrose, red colorant, fructose, NbN evocation (heirloom tomato flavor, roasted chicken flavor), salt	Heirloom tomato essence was mixed with red colorant, salt, fructose and xanthan gum. It was then frozen in a spherical mold. Rice soufflé was prepared from starch, milk, xanthan gum, sucrose, dextrose and egg whites. Finally, the tomato gel was added to the mixture, steamed in a silicone mold and drizzled with chicken essence.

**Table A2:** Statements from the Food Neophobia Scale and Domain-Specific Innovativeness used for research.

To What Extent Do You Agree with the Following Statements (Please Mark X in the Appropriate Box)?		Strongly Disagree	Disagree	More or Less Disagree	Undecided	More or Less Agree	Agree	Strongly Agree
		1	2	3	4	5	6	7
1	I am constantly sampling new and different cuisines. (R)							
2	I do not trust new foods.							
3	If I do not know what is in a food, I will not try it.							
4	I like foods from different countries. (R)							
5	Modernist foods look too weird to eat.							
6	At dinner parties I will try a new food. (R)							
7	I am afraid to eat things I have never had before.							
8	I am very particular about the foods I will eat.							
9	I will eat almost anything. (R)							
10	I like to try restaurants with new cuisines. (R)							
11	I buy new foods before other people do							
12	In general, I am among the first in my circle of friends to buy new foods							
13	Compared to my friends I buy more new foods							
14	Even though new foods are available in the store, I do not buy them (R)							
15	In general, I am the last in my circle of friends to know the trademarks of new foods (R)							
16	I will not buy new foods, if I have not tasted them yet (R)							

R—reversed; Questions 1–10. Adapted Food Neophobia Scale by Pilner and Hobden (1992); Questions 11–16. Domain-Specific Innovativeness Scale by Goldsmith and Hofacker (1991) with modifications of Huotilainen et al. (2006).
